# *Escherichia coli* as a Multifaceted Pathogenic and Versatile Bacterium

**DOI:** 10.3389/fcimb.2020.548492

**Published:** 2020-12-21

**Authors:** Vânia Santos Braz, Karine Melchior, Cristiano Gallina Moreira

**Affiliations:** Department of Biological Sciences, School of Pharmaceutical Sciences, São Paulo State University (UNESP), Araraquara, Brazil

**Keywords:** treatment, genetic mobility, pathogenesis, *Escherichia*, multiresistant

## Abstract

Genetic plasticity promotes evolution and a vast diversity in *Escherichia coli* varying from avirulent to highly pathogenic strains, including the emergence of virulent hybrid microorganism. This ability also contributes to the emergence of antimicrobial resistance. These hybrid pathogenic *E. coli* (HyPEC) are emergent threats, such as O104:H4 from the European outbreak in 2011, aggregative adherent bacteria with the potent Shiga-toxin. Here, we briefly revisited the details of these *E. coli* classic and hybrid pathogens, the increase in antimicrobial resistance in the context of a genetically empowered multifaceted and versatile bug and the growing need to advance alternative therapies to fight these infections.

## Introduction

*Escherichia coli* (or *E. coli*) is a Gram-negative versatile bacterium, easily found and amenable to natural and random genetic alteration. There is a vast collection of sequenced *E. coli* genomes which exhibit different sizes and genomic diversity among commensal and pathogens, indicating a great assortment within the same bacterial species. They comprise of non-pathogenic bacteria that may act as commensals and belong to the normal intestinal microbiota of humans and many animals. There are also pathogenic variants, divided as diarrheagenic and extraintestinal pathogens, with different pathotypes and various natural hybrid strains (**Tables 1** and **2**). These variants can be facultative or obligate pathogens. The facultative bacteria are part of the intestinal tract and may act as opportunistic pathogens when outside of their natural habitat, causing various types of extraintestinal infections. On the other hand, intestinal obligate pathogenic variants cause infections in distinct conditions, from moderate diarrhea to more threatening cases, as lethal outcome (Kaper et al., 2004; Köhler and Dobrindt, 2011).

**Table 1:** Classic *E. coli* pathotypes main features: extraintestinal (ExPEC) and diarrheagenic (DEC).

*E. coli* Pathotype (DEC and ExPEC)	Main virulence traits	Clinical manifestation	Antimicrobial resistance (AMR) commonly found	Mobile genetic resistance determinants	References
Shiga toxin-producing (STEC)	Shiga-toxin	Not associated with human diseases	Streptomycin, Ampicillin, Tetracyclin and sulphonamides	ND	Jerse et al., 1990; Kaper et al., 2004; Day et al., 2017
Enterohemorrhagic (EHEC)	EscF, EscC, EspA, EspB, EspD, Intimin, Tir, and Shiga-toxin	Foodborne Bloody diarrhea and HUS	Streptomycin, Ampicillin, Tetracyclin and sulphonamides	Resistance plasmid-mediated (as pO157, pO111-CRL115, pO26-CRL125, pO145-13514)	Knutton et al., 1989; Mellies et al., 1999; Kaper et al., 2004; Garmendia et al., 2005; Day et al., 2017
Enteropathogenic (EPEC)	EscF, EscC, EspA, EspB, EspD, Intimin, Tir, EAF plasmid (tEPEC) and Bfp (tEPEC)	Watery diarrhea	Streptomycin, Ampicillin, Tetracyclin, Trimethoprim and Sulfamethoxazole	Resistance plasmid-mediated (as pEAF, MB80, pB171_90, pED208)	Tobe et al., 1999; Trabulsi et al., 2002; Kaper et al., 2004; Ingle et al., 2018
Enteroaggregative (EAEC)	pAA plasmid, aggregated fimbriae adhesion (AAF), AggR regulator and dispersin	Acute and chronic diarrhea	Ampicillin, Trimethoprim, Sulfamethoxazole, Nalidixic acid, and ciprofloxacin	Resistance plasmid-mediated (as pAA), chromosomal *gyrB* and *parC* mutations	Regua-Mangia et al., 2009; Aslani et al., 2011; Gomes et al., 2016; Pawłowska and Sobieszczańska, 2017; Chattaway et al., 2017
Enteroinvasive (EIEC)	Plasmid pINV and invasins	Bacillary Dysentery	Carbapenem, fosfomycin-trometanol, nitrofurantoin, chloramphenicol, β-lactams, nalidixic acid, ampicillin and fluoroquinolones	Resistance plasmid-mediated, chromosomal *gyrB* and *parC* mutations	Kaper et al., 2004; Baylis et al., 2006; Gomes et al., 2016; Pawłowska and Sobieszczańska, 2017
Enterotoxigenic (ETEC)	Thermostable (ST) and thermolabile (LT) enterotoxins	Watery diarrhea, known as traveler’s diarrhea	Ampicillin, sulfamethoxazole, tetracycline and azithromycin	Resistance plasmid-mediated (distinct Inc type conjugative plasmids)	Kaper et al., 2004; Medina et al., 2015; Gomes et al., 2016; Pawłowska and Sobieszczańska, 2017
Diffusely-adhering (DAEC)	Afa/Dr adhesins	Acute diarrhea to assypmtomatic cases	Ampicillin, Trimethoprim, Sulfamethoxazole, Fosfomycin, piperacillin, tetracycline, ciprofloxacin, co-trimoxazole, nitrofurantoin, oxacillin, bactericin, cloxacillin, chloramphenicol, and nalidixic acid	Resistance plasmid-mediated, chromosomal *gyrB* and *parC* mutations	Kaper et al., 2004; Nash et al., 2010; Servin, 2014; Gomes et al., 2016
Adherent-invasive (AIEC)	type VI secretion system, type I pili, long polar fimbriae	Chronic gut inflammation and Crohn´s disease	Ampicillin and ciprofloxacin	Resistance plasmid-mediated, chromosomal *gyrB* and *parC* mutations	Kaper et al., 2004; Nash et al., 2010; Barrios-Villa et al., 2018
Cell-detaching (CDEC)	K-hemolysin, pyelonephritis-associated pili and cytotoxic necrotizing factor 1 (CNF1)	Diarrhea in infants, cell detaching, and inked to Crohn´s disease cases	Amoxicillin-clavulanic acid, ampicillin, mezlocillin, piperacillin, tetracycline, trimethoprim, trimethoprim-sulfamethoxazole, spectinomycin, streptomycin and sulfonamide	Resistance plasmid-mediated, integrons	Elliott et al., 1998; Fábrega et al., 2002; Okeke et al., 2002; Kaper et al., 2004; Rakitina et al., 2017
Uropathogenic (UPEC)	P fimbriae, certain other mannose-resistant adhesins, and type 1 fimbriae, K capsule, Hemolysin, Aerobactin	Urinary and Bloodstream infections	Fluoroquinolone, aminoglycosides, trimethoprim-sulfamethoxazole and carbapenems	Resistance plasmid-mediated, transposons, integrons, chromosomal *gyrA, gyrB, parE*, *parC* and *marA* mutations	Kaper et al., 2004; Mobley et al., 2009; Petty et al., 2014
Sepsis-causing (SEPEC)	Type 1, P, and S fimbriae, K capsule K1/K5, hemolysin, aerobactin, yersiniabactin, salmochelin, CNF1, secreted autotransporter toxin, serum resistance, and colicin V	Bacteremia and sepsis	Carbapenems	Resistance plasmid-mediated, integrons	Kaper et al., 2004; Mokady et al., 2005; Nagarjuna et al., 2018
Neonatal meningitis-associated (NMEC)	*ompTp*, *hlyF*, *cvaC*, *etsA*, *cvaA*, *etsB*, *cvaB*, *iss*, *iutA*, and *tsh*	Meningitis, and bacteremia in neonates	Streptomycin sulfisoxazole, ampicillin, tetracycline, chloramphenicol, kanamycin and trimethoprim-sulfamethoxazole	Resistance plasmid-mediated	Korhonen et al., 1985; Kaper et al., 2004; Logue et al., 2012

ND, non-described.

**Table 2:** Hybrid pathogenic (HyPEC) main features described.

HyPEC	Main features	Hybrid virulence traits identified	Clinical manifestation	Antimicrobial resistance (AMR) described	References
O1O4:H4 EAEC/STEC	Hybrid EAEC with STEC	Aggregative typical fimbriae, Shiga toxin	Diarrhea, HUS	Quinolones and β-lactams	Bielaszewska et al., 2011; Rasko et al., 2011; Muniesa et al., 2012; Navarro-Garcia, 2014; Ribeiro et al., 2019
O80:H2 STEC/ExPEC	Hybrid STEC with ExPEC	Intimin, Shiga toxin and pS88-like plasmid	HUS, Bacteremia	β-lactams	Peigne et al., 2009; Mariani-Kurkdjian et al., 2014
O2:H6 STEC/UPEC	Hybrid STEC with UPEC	*α-hlyA*, *cnf1* and *clb* genes	Diarrhea, Urinary tract infections, HUS	ND	Bielaszewska et al., 2014
ST131 UPEC/EAEC	Hybrid ExPEC with EAEC	pAA plasmid	Urinary infections, Bloodstream infections and Diarrhea	β-lactams	Boll et al., 2018
EPEC/ETEC	Hybrid EPEC and ETEC	Intimin, LEE island, ST and LT toxin	Diarrhea, Mild fever and cough	β-lactams, SUT, and quinolones	Dutta et al., 2015
STEC/ETEC	Hybrid STEC and ETEC	Intimin, Shiga toxin (Stx2) and LT toxin	Acute diarrhea and HUS	ND	Lindstedt et al., 2018
O137:H6 (ST2678) EPEC/STEC	Hybrid EPEC and STEC	Intimin, BFP, Shiga toxin (Stx2) and AIDA-I autotransporter	Diarrhea and HUS	ND	Gioia-Di Chiacchio et al., 2018
STEC/ETEC	Hybrid STEC and ETEC	Intimin, Shiga toxin, ST and LT toxins	Acute diarrhea and HUS	ND	Nyholm et al., 2015
EPEC/ETEC	Hybrid EPEC and ETEC	Intimin, BFP, ST and LT toxins	Diarrhea, Mild fever and cough	ND	Hazen et al., 2017

HyPEC, Hybrid Pathogenic E. coli; HUS, Hemolytic Uremic Syndrome; ND, non-described; SUT, Trimethoprim-sulfamethoxazole.

*E. coli* pangenome studies indicate enormous capacity to evolve by gene acquisition and genetic modification. Besides, these genomes have a mosaic-like structure consisting of a core genome, encoding essential cellular functions, and an accessory genome with flexible strain-specific sequences. Thus, *E. coli* is a model well established for studying the interdependence of genome architecture and the lifestyle of bacteria (Touchon et al., 2009; Dobrindt et al., 2010).

Based on virulence factors in *E. coli* genomes and phenotypic traits, the human pathotypes of diarrheagenic *E. coli* (DEC) are differentiated from non-pathogenic *E. coli* and extraintestinal pathogenic *E. coli* (ExPEC). The ExPEC are classified as uropathogenic *E. coli* (UPEC), sepsis-causing *E. coli* (SEPEC) and neonatal meningitis-associated *E. coli* (NMEC) (Kaper et al., 2004). Recent pathogenomics and phenotypic classification have revisited the DEC group as nine distinct pathotypes, proposed by their differential features and the essential virulence genes defining each subgroup, such as Shiga toxin-producing *E. coli* (STEC), enterohemorrhagic *E. coli* (EHEC), enteropathogenic *E. coli* (EPEC), enterotoxigenic *E. coli* (ETEC), enteroinvasive *E. coli* (EIEC), enteroaggregative *E. coli* (EAEC), diffusely-adhering *E. coli* (DAEC), adherent-invasive *E. coli* (AIEC), and cell-detaching *E. coli* (CDEC) (Kaper et al., 2004; Pawłowska and Sobieszczańska, 2017) (**Table 1**).

Herein, we briefly describe the diversity of these classic and novel emerging *E. coli* pathotypes and their genetic plasticity in a multifaceted organism. The mobile genetic elements are responsible for the appearance of novel hybrid strains with distinct assortment of virulence and antimicrobial resistance traits, bringing up the urgent need to reconsider the forms of treatment for these infections.

## Types of *E. coli*: Many Flavors Within a Single Bacterial Species

*E. coli* is one of the most genetically versatile microorganisms and is able to colonize and persist in several niches, both in the environment or in hosts. Commensal *E. coli* strains colonize the gastrointestinal tract of humans a few hours after birth, resulting in a symbiotic relationship between the microbiota and its host (Ducarmon et al., 2019). However, the mechanisms by which *E. coli* ensures this efficient symbiosis is not well known. It could be related to its high ability to use nutrients in the colon (Fabich et al., 2008; Ducarmon et al., 2019). Several studies have shown that competition for nutrients between microbiota and pathogens limits the colonization of the pathogens, leading to fierce competition among these microorganisms (Lustri et al., 2017).

Occasionally, pathogenic *E. coli* cannot be distinguished from commensal *E. coli*, only based on specific virulence factors, as some previously described in ExPEC strains (Köhler and Dobrindt, 2011). However, this scenario is changing due to sophistication and availability of molecular typing methodologies. New computational approaches bring countless important information about host-pathogen relationships, reservoir, clinical diagnoses, and novel ExPEC transmission pathways (Johnson and Russo, 2018). Often, virulence genes are located in transmissible genetic elements such as genomic islands, bacteriophages, insertion sequences (ISs), integrons, plasmids, and transposons; hence, they can be easily exchanged among different bacteria (Hacker et al., 2003; Dobrindt et al., 2010). They also carry multiple antibiotic resistance genes that have been under strong selective pressure as consequence of the extensive use of antibiotics (Brzuszkiewicz et al., 2009).

Common genetic changes in *E. coli* genomes ensure high diversity due to the gain and loss of genes through genetic modification events. There are many strains of ExPEC that normally colonize the gut asymptomatically, as members of the intestinal microbiota. Nonetheless, only a subset of ExPEC as UPEC, SEPEC and NMEC are responsible for the vast majority of infections such as urinary tract infections, sepsis, and meningitis (Kaper et al., 2004). There is a great variety of virulence factors in ExPEC strains, such as adhesins (fimbrial and non-fimbrial), siderophores, toxins, invasins, the ability to survive in serum, among others. Moreover, many of these virulence factors may occur combined within the same strain and act synergistically. Despite extra factors, the septic strains always possess at least an adherence system, an iron uptake system and genes for serum survival (Biran and Ron, 2018; Johnson and Russo, 2018) (**Table 1**).

The genetic evolution in *E. coli* pathogenesis employs horizontal transfer mechanisms within same and across similar species. Therefore, the IS, transposons and integrons may facilitate novel rearrangements within the genome, such as duplication and suppression of genes and also capture of new genes. This genetic material transit can result in greater flexibility concerning various features, such as the transition of pathogenic bacteria between humans and animals, resistance to antimicrobials, appearance of emerging pathogens due to the gain of virulence genes, increased pathogenicity, among other features (Frost et al., 2005; Brigulla and Wackernagel, 2010; Dobrindt et al., 2010; Jackson et al., 2011; Sheppard et al., 2018). All these conditions may contribute to the virulence of these bacteria, like the bacteriophage importance in the pathogenesis. The horizontal transfer between different strains favors the emergence of new pathogenic strains with discrepancies in the bacteriophage repertoire affecting directly their virulence (Manning et al., 2008; Ogura et al., 2009; Dobrindt et al., 2010; Jackson et al., 2011).

The co-evolution of bacterial genomes with plasmids, besides potential genetic and phenotypic gain may impact cellular metabolism to ensure the maintenance and stability of the plasmid (Jackson et al., 2011). Many ExPEC virulence genes are encoded within plasmids, often belonging to the ColV family, which encodes colicin, serum survival factors and iron uptake systems (Biran and Ron, 2018). Similarly, intestinal pathogens carry a variety of types of plasmids, associated with virulence, majorly belonging to the incompatibility group IncF, which has transfer functions (Carattoli, 2009). There are virulence plasmids essential for some pathotypes of *E. coli*, such as pINV and pAA, respectively, in EIEC and EAEC, according to each own group features (Kaper et al., 2004).

Although, all ExPEC and DEC pathotypes are not enough to fully classify all pathogenic *E. coli* strains, since these bacteria are so variable, allowing constant appearance of distinct hybrid-formed strains within this dynamic bacterial species. The carriage of virulence genes essential to the pathogenesis of each pathotype and the ability to adapt to different conditions allow the emergence of hybrid pathogenic *E. coli* (HyPEC).

## Genetic Plasticity and Emergent *E. coli* Pathogen: HyPEC

*E. coli* has an astonishing facility to amend very well, replicate and disseminate. These features allowed the advent of novel HyPEC. Acquired virulence genes and novel functions appear from mutation, recombination and other genetic changes. All these genetic differences have increased the occurrence of novel hybrid and antimicrobial resistance among DEC and ExPEC (Dobrindt et al., 2003; Bielaszewska et al., 2007; Khan et al., 2018).

Recently, a HyPEC strain received widespread attention after an outbreak of foodborne bloody diarrhea and hemorrhagic uremic syndrome (HUS) in Germany. This outbreak of *E. coli* O1O4:H4 was associated with consumption of raw fenugreek sprouts, as a hybrid EAEC strain with STEC features, like Shiga toxin presence. This HyPEC was quickly sequenced and unraveled its intricate nature, but even with a quick response and identification it was not enough to avoid 3,842 hospitalizations with many fatalities in Europe and North Africa (Bielaszewska et al., 2011; Rasko et al., 2011). Emerging processes are responsible for the HyPEC occurrences. Herein, the combined enteroaggregative features in a rare serotype was responsible to high attachment to cells and a biofilm formation (Navarro-Garcia, 2014; Ribeiro et al., 2019). Moreover, this strain has gained *stx2* gene lambdoid phage integrated in the genome, thus it may release the Shiga-toxin. These features have increased HUS occurrence during the outbreak on this HyPEC when compared to STEC (Muniesa et al., 2012).

Many distinct genetic hybrid examples are reported in *E. coli*, such as STEC/ExPEC O80:H2 serotype, which caused HUS and bacteremia due the presence of *stx2* and *eae* genes from STECs and pS88-like plasmid, described in meningitis, urosepsis and avian pathogenic strains of ExPEC (Peigne et al., 2009; Mariani-Kurkdjian et al., 2014). The STEC/UPEC strain O2:H6 serotype, a STEC with virulence genes as *α-hlyA*, *cnf1*, and *clb* from UPEC that have ability to cause diarrhea and urinary tract infections (Bielaszewska et al., 2014). The EPEC/ETEC strain has acquired the LEE island and encodes the LT toxin (Dutta et al., 2015). The broadly reported multidrug resistant *E. coli* ST131 is example of highly virulent ExPEC associated with urinary and bloodstream infections. It has also acquired enteroaggregative diarrheagenic phenotype due to pAA plasmid presence (Boll et al., 2018). Many others HyPEC are described as case report, but not fully characterized. Here, we have briefly sampled some of the acquired genes by these strains, their direct impact in virulence and their hybrid nature (**Table 2**). Comparable to these HyPEC, the coined terms hybrid- and hetero-pathogenic *E. coli* have been recently described as new combination of virulence factors among classic *E. coli* groups. Together, they show differences between typical and atypical subgroups within the EAEC and EPEC pathotypes and hybrids, such as EPEC/STEC, ExPEC/EPEC and ExPEC/EAEC hybrids (Santos et al., 2020). Similar to our approach here, this study shows how this topic is critical in the field.

The high prevalence of classic pathogenic *E. coli* and appearance of HyPEC occur *via* similar genetic mechanisms, which also enable bacteria to resist the presence of distinct antimicrobials. Bacteria resistant to various classes of antibiotics are related to the complex combination of intrinsic and acquired resistance genes, which may act synergistically (Cag et al., 2016; Khan et al., 2018). Together that brings multiresistant bacteria, as an alarming factor reported worldwide in several bacterial species. WHO has prioritized studies on AMR bacteria, including Enterobacteriaceae, based on recent surveillance reports (WHO, 2018).

## Emerging Hybrids and Alternative Therapies

The complex combination of multidrug-resistant bacteria and emerging hybrid bacteria with intrinsic or acquired bacterial virulence factors disseminated by genetic mobility elements, the intense and inappropriate use of antibiotics have simultaneously favored the emergence of resistance to various antibiotics (Khan et al., 2018). That is a special challenge to these hybrid strains, since these HyPEC gathered virulence traits and acquired antibiotic resistance, together these points raise the importance to alternative treatments. These options are crucial to reduce the use of antibiotics and the consequent increase of antimicrobial resistance. Novel therapies are urgent to replace prophylactic and treatment with antibiotics by probiotics, prebiotics, enzymatic compounds, vaccines, monoclonal antibodies, phage therapy, antivirulence compounds, among other possibilities (Gadde et al., 2017).

Recently, different vaccine strategies have been used for pathogenic *E. coli* infection as an alternative to antibiotic therapy (Rojas-Lopez et al., 2018), including vaccines with attenuated toxins (McKenzie et al., 2007; Bitzan et al., 2009), attenuated bacterial cell (Calderon Toledo et al., 2011), individual components of virulence factors such as Shiga toxin (Liu et al., 2009), EspA or Intimin (Oliveira et al., 2012), small peptides (Zhang et al., 2011), DNA (García-Angulo et al., 2014) or polysaccharides (Ahmed et al., 2006; van den Dobbelsteen, 2016), as well detailed in the literature. Commercial vaccines have aimed the use to protect livestock, such as poultry, swine and bovine herds, against respectively to APEC, like Poulvac® *E. coli*, ETEC and EHEC infections (Sadeyen et al., 2015; Nesta and Pizza, 2018). Vaccines with a modern approach and technology still are a promising strategy to protect against emergent HyPECs infections in humans and livestock.

Recent studies have revisited the phage therapy as a biological alternative, which employs strictly lytic phages uncapable of lysogenization (Carter et al., 2012). Studies have demonstrated ability of phages to decrease biofilm formation in UPEC (Chibeu et al., 2012), increased mice rate survival in *E. coli*-induced pneumonia (Dufour et al., 2015). Moreover, lytic bacteriophages were used to infect and kill bacteria harboring phage-dependent conjugative plasmid to avoid emergence of multiresistant bacteria (Ojala et al., 2013; Tagliaferri et al., 2019). The phages cocktail EcoShield™ is already commercialized (Intralytix) and it has been reported to significantly reduce the *E. coli* O157:H7 contamination on surfaces and food (Abuladze et al., 2008; Carter et al., 2012). Additionally, mutual use of phages with antibiotics have emerged, with SPR02 and DAF6 phages combined with enrofloxacin have shown promising data, rescuing chickens challenged with avian pathogenic *E. coli* infection (Tagliaferri et al., 2019).

The novel approach *via* antivirulence-directed compounds works disarming the pathogens’ ability to cause disease by inhibiting their virulence factors, favoring the host’s immune defenses during the bacterial clearance. These compounds do not induce bacterial resistance as antibiotics, because they disarm the pathogen, instead of directly targeting its growth. Therefore, as they are directed to specific factors for pathogenesis, they potentially reduce the selection of resistance and limit collateral damage to the microbiota. Some virulence inhibitors are effective against many pathogens, molecules such as LED209, HC102A, HC103A, Artemisinin, and Ethoxzolamide, by inhibit different two-component systems as QseBC in *E. coli* and other enteropathogens (Sperandio et al., 2003; Rasko et al., 2008; Yang et al., 2014; Xue et al., 2015; Kim et al., 2020), Bicyclic 2-pyridones, Biaryl mannoside, Nitazoxanide and FN075, avoiding the initial bacterial adhesion; and compounds like Toxtazins A and B, Ebselen, 7086, 7812, 7832, BPT15, and BBH7, blocking toxins and secretion systems (Payne, 2008; Johnson and Abramovitch, 2017).

## Conclusion

The forces that shape the evolution in *E. coli* comprise vast repertoire, affecting genetic flexibility and excessive permissiveness to acquire and donate DNA *via* horizontal gene transfer. These features guarantee the spread of antibiotic resistance as well as virulence factors inherited among the various pathotypes of *E. coli.* The exact identification and assessment assist researchers to better understand this bacterium modification, diagnosis, public health and treatment. *E. coli* strains with multiple and distinct factors are probably very common but unreported, since these *E. coli* strains have developed many strategies to persist in different settings and successfully infect the host. These strategies result in an immense variety of microorganisms, ranging from avirulent to extremely virulent strains that can cause intestinal or extraintestinal diseases. *E. coli* strains have great potential for dissemination and capacity to pass along hereditary elements. Currently, these HyPEC strains are a very concerning threat that demands more studies and the development of novel treatment methods.

## Author Contributions

VB: writing and organization. KM: writing. CM: writing and mentoring. All authors contributed to the article and approved the submitted version.

## Funding

Financially supported by FAPESP (grants 2014/06779-2, 2018/22412-2, 2018/22042-0, and 2019/03049-7), CNPq (307418/2017-0), and “Programa de Apoio ao Desenvolvimento Científico da Faculdade de Ciências Farmacêuticas da UNESP-PADC. This study was financed in part by the Coordenação de Aperfeiçoamento de Pessoal de Nível Superior - Brasil (CAPES) - Finance Code 001.

## Conflict of Interest

The authors declare that the research was conducted in the absence of any commercial or financial relationships that could be construed as a potential conflict of interest.
